# *MiR-506* Targets *UHRF1* to Inhibit Colorectal Cancer Proliferation and Invasion via the *KISS1/PI3K/NF-*κ*B* Signaling Axis

**DOI:** 10.3389/fcell.2019.00266

**Published:** 2019-11-15

**Authors:** Yilin Lin, Zhihua Chen, Yan Zheng, Yisu Liu, Ji Gao, Suyong Lin, Shaoqin Chen

**Affiliations:** ^1^Department of Gastrointestinal Surgery, The First Affiliated Hospital of Fujian Medical University, Fuzhou, China; ^2^School of Nursing, Fujian Medical University, Fuzhou, China

**Keywords:** CRC, *miR-506*, *UHRF1*, proliferation, invasion

## Abstract

**Background:**

The *UHRF1* gene is an epigenetic modification factor that mediates tumor suppressor gene silencing in a variety of cancers. Related studies have reported that *UHRF1* can inhibit the expression of the *KISS1* gene. However, the regulatory mechanism underlying *UHRF1* expression in colorectal cancer (CRC) is still unclear. The aim of this study was to gain a better understanding of the regulation of *UHRF1* expression in CRC and to determine whether it regulates the mechanism by which *KISS1* promotes CRC metastasis.

**Methods:**

In the present study, the levels of *miR-506*, *UHRF*1 and *KISS1* expression in CRC tissues and in human CRC cell lines were studied using quantitative real-time PCR (qRT-PCR) and Western blotting. Cell proliferation, migration, and invasion assays are used to detect cell proliferation, migration, and invasion. A dual-luciferase reporter system was used to confirm the target gene of *miR-506*.

**Results:**

This study found that *UHRF1* protein is highly expressed in CRC tissues and negatively correlated with *KISS1* protein expression. *UHRF1* overexpression activates the *PI3K/NF-*κ*B* signaling pathway by inhibiting the mRNA expression levels of pathway mediators. Bioinformatics analysis and luciferase reporter gene assays confirmed that *miR-506* targets *UHRF1*.

**Conclusion:**

This study identified the regulation of *UHRF1* expression in CRC and the mechanism of CRC metastasis. *UHRF1* may be a new potential target molecule for future CRC metastasis treatment.

## Introduction

Colorectal cancer (CRC) is a common malignant tumor of the digestive tract. Globally, the incidence of CRC ranks third among malignancies, below lung cancer and breast cancer (Bray et al., 2018). The lack of typical clinical symptoms is one of the reasons for the low rate of early CRC diagnosis. Comprehensive treatments such as surgery, radiation therapy, chemotherapy, biological targeting, and immunotherapy are currently the standard treatment approaches for CRC (Ni et al., 2018; Ganesh et al., 2019; Li S. et al., 2019; Siravegna et al., 2019). However, CRC metastasis remains an urgent problem, as metastasis negatively affects patient prognosis. Gene-targeted therapy has great potential, and finding effective therapeutic targets is the focus of current research (de Mel et al., 2019; Erel-Akbaba et al., 2019; Schiza et al., 2019). Studies have shown that the occurrence and development of CRC involve the activation of proto-oncogenes and the inactivation of tumor suppressor genes (Sanz-Garcia et al., 2017; Zhang and Shay, 2017), as well as microRNA changes in the tumor microenvironment (Vu and Datta, 2017; De Robertis et al., 2018; Lin X. et al., 2019; Yu et al., 2019).

The *UHRF1* (ubiquitin-like with plant homeodomain and RING finger domains 1) gene is an epigenetic modification factor (Harrison et al., 2016; Xie and Qian, 2018). Studies have shown that *UHRF1* recognizes hemi-methylated DNA, which appears at DNA replication forks, and assists *DNMT1* in DNA methylation (Lu and Wang, 2013; Ferry et al., 2017). A large number of studies have shown that *UHRF1* is highly expressed in a variety of malignant tumor tissues, including breast cancer, bladder cancer, and prostate cancer (Geng et al., 2013; Ying et al., 2015; Wan et al., 2016; Li J. et al., 2019) and that it is involved in tumorigenesis and cancer progression (Alhosin et al., 2011, 2016). In addition, *UHRF1* can inhibit cell apoptosis through the *ROS*-related signaling pathway in gastric cancer (Zhang et al., 2018), and *UHRF1* was found to enhance the invasive ability of tumor cells through the *Keap1-Nrf2* pathway in pancreatic cancer (Abu-Alainin et al., 2016). A recent study found that *UHRF1* silencing can inhibit retinoblastoma proliferation and promote apoptosis through the *PI3K/AKT* signaling pathway (Liu et al., 2019). Studies have found that the expression of *UHRF1* in CRC is related to the depth of invasion of the tumor and that knocking down the expression of *UHRF1* can inhibit the proliferation of CRC cells (Kofunato et al., 2012). Additionally, *UHRF1* silences *PPARG* expression and mediates the progression of CRC (Sabatino et al., 2012). Furthermore, *UHRF1* may promote CRC growth and metastasis by inhibiting p16 (ink4a) (Wang et al., 2012). Ashraf et al. (2017) highlighted the deregulation of *UHRF1* in various cancers, including CRC, and its prognostic value in cancers. This highlights *UHRF1* dysregulation and the importance of identifying different strategies to target *UHRF1* in cancers, as well as the prognostic value of *UHRF1* (Ashraf et al., 2017). Therefore, *UHRF1* may be an important biomarker in the diagnosis, treatment, and prognosis of CRC.

*KISS1* was first discovered in melanoma; subsequently, *KISS1* was reported to affect the growth, invasion, and migration of tumor cells and confirmed to be an important tumor suppressor gene in multiple types of malignant tumors (Manley et al., 2017; Liu et al., 2018; Platonov et al., 2018). Suppression of *KISS1* expression is closely related to DNA methylation in CRC tissues (Chen et al., 2014), while *KISS1* overexpression has been reported to inhibit the invasion of CRC cells by blocking *PI3K/AKT* signaling (Chen et al., 2016; Chipman and Pasquinelli, 2019). Studies have also shown that overexpression of *UHRF1* can inhibit the expression of *KISS1* mRNA in bladder cancer (Zhang et al., 2014). However, whether *UHRF1* can inhibit *KISS1* and activate the *PI3K/NF-*κ*B* signaling pathway in CRC remains unclear.

MicroRNAs are a class of non-coding RNAs that are abundantly found in various organisms ranging from viruses to humans. They are approximately 22 nucleotides in length. One of the functions of miRNAs is to bind to the 3′-non-coding regions of target mRNAs [3′ untranslated region (3′UTR)] to inactivate the genes (Chipman and Pasquinelli, 2019). Studies have found that at least one-third of protein-coding genes are regulated by miRNAs, including those involved in cellular differentiation, proliferation, metabolism, apoptosis, and migration (Farazi et al., 2013; Hayes et al., 2014). Studies have found that *miR-501-3p* promotes CRC progression via activation of *Wnt/*β catenin signaling (Wu et al., 2019), that *miR-4319* suppresses CRC progression by targeting *ABTB1* (Huang et al., 2019), and that *miR-144* suppresses aggressive phenotypes of tumor cells by targeting *ANO1* in CRC (Jiang et al., 2019). These studies have shown that miRNA plays an important role in CRC. Previous studies have found that *miR-202* inhibits CRC proliferation and invasion by targeting *UHRF1* (Lin Y. et al., 2019). *MiR-9* targets *UHRF1* and inhibits the proliferation and apoptosis of CRC cells (Zhu et al., 2015). Choudhry et al. (2018) reported the importance of the miRNA/*UHRF1* strategy for targeting various cancers. The study revealed the importance of miRNA therapy targeting *UHRF1*, particularly in CRC. Therefore, it is important to identify miRNAs that target *UHRF1* and to study their mechanisms of action in cancer. *MiR-506* is located on the X chromosome and is a member of the *miR-506-514* sequence family (Bentwich et al., 2005). Increased expression of *miR-506* inhibits tumor cell proliferation and promotes tumor cell senescence and apoptosis, and it has been reported to exert anticancer effects in ovarian cancer, breast cancer, and liver cancer (Wang et al., 2014; Sun et al., 2015; Yu et al., 2015). However, studies have also confirmed that *miR-506* acts as a carcinogenic factor in melanoma (Streicher et al., 2012). At present, *miR-506* has been reported to be differentially expressed in different tumors and to play oncogenic or tumor-suppressive roles in different tumors. To date, the expression and mechanism of *miR-506* in CRC remains unclear.

This study demonstrates that *UHRF1* activates the *PI3K/AKT/NF-*κ*B* signaling pathway by inhibiting *KISS1* mRNA expression in CRC. Furthermore, *miR-506* targets *UHRF1* via the *KISS1/PI3K/NF-*κ*B* signaling axis to inhibit CRC proliferation, migration, and invasion both *in vivo* and *in vitro*. Our findings provide new insights into the underlying mechanisms of *UHRF1* in CRC and provide potential therapeutic targets for the treatment of CRC.

## Materials and Methods

### Human Tissues

A total of 121 CRC tissues and 121 adjacent normal tissues were collected from the First Affiliated Hospital of Fujian Medical University in 2017 and 2018. All tissue samples were immediately frozen in liquid nitrogen for histological examination. Tumor burden was determined using the American Joint Committee on Cancer TNM staging system. Patients provided informed consent for the use of human materials in the study, which was approved by the Ethics Committee of Fujian Medical University.

### Immunohistochemical Staining and Analysis

Immunohistochemistry was performed on 4-μm-thick paraffin-embedded sections of both CRC tissues and adjacent normal tissues. Sections were stained to determine the expression levels of the *UHRF1* and *KISS1* proteins. The slides were incubated overnight at 4°C with an anti-*UHRF1* antibody (Sigma, United States) or anti-*KISS1* antibody (Sigma, United States) diluted 1:200. After incubation, the slides were washed with phosphate-buffered saline (PBS) and incubated with a fluorescein isothiocyanate-conjugated goat anti-mouse IgG secondary antibody (ZSGB-BIO, Beijing, China) for 30 min. The slides were washed with PBS and then mounted with anti-fade reagent (Invitrogen, Carlsbad, CA, United States). Finally, the stained slides were observed using an Olympus CX41 fluorescence microscope (Olympus, Tokyo, Japan). The stained tumor sections were examined for positively stained tumor cells and the intensity of immunohistochemical signals and scored independently by two observers. According to the proportion of positively stained tumor cells, the sections were scored as follows: (0) no positive tumor cells; (1) <10% positive tumor cells; (2) 10–50% positive tumor cells; and (3) >50% positive tumor cells. The staining intensity was graded according to the following criteria: (0) no staining; (1) weak staining (light yellow); (2) moderate staining (yellow brown); and (3) strong staining (brown). A total score of > 3 points was considered high expression, and ≤ 3 points was considered low expression.

### Detection of *miR-506* and *UHRF1* mRNA Expression in Human Specimens

Human tissue specimens were ground into a powder using liquid nitrogen. Tissue RNA was extracted with TRIzol reagent (TransGen Biotech, Beijing, China), and then, *miR-506* and *UHRF1* mRNA expression levels were analyzed by quantitative real-time PCR (qRT-PCR). The qRT-PCR assay was performed as follows: RNA was detected using a reverse transcription kit (TaKaRa, Dalian, China) and an amplification kit (TaKaRa) following the manufacturer’s instructions. *U6* was used as the internal control for *miR-506* expression levels, and *GAPDH* was used as the internal control for the *UHRF1* gene. The reaction mixture contained 10 μl of SYBR Premix Ex Taq 2, 1 μl of each primer, 2 μl of the cDNA template, and 6 μl of ddH_2_O for a final volume of 20 μl. The thermal cycling parameters for amplification were as follows: a denaturation step at 95°C for 30 s, followed by 40 cycles at 95°C for 5 s and a final holding step at 60°C for 34 s. Relative gene expression was evaluated with Data Assist software version 3.0 (Applied Biosystems, Foster City, CA, United States). The relative expression levels were determined according to the 2^–Δ^ ^Δ^ ^CT^ method. The assays were performed in triplicate. Primer sequences for each gene were shown in Table 1.

**TABLE 1:** Primer sequences for each gene.

**Gene**	**Primer sequence**	
*UHRF1*	Forward	5′-CGACGGAGCGTACTCCCTAG-3′
	Reverse	5′-TCATTGATGGGAGCAAAGCA-3′
*GAPDH*	Forward	5′-CCCTTCATTGACCTCAACTACATG-3′
	Reverse	5′-TGGGATTTCCATTGATGACAAGC-3′
*U6*	Forward	5′-CGCTTCGGCAGCCACATATACTA-3′
	Reverse	5′-CGCTTCACGAATTTGCGTGTCA-3′
*KISS1*	Forward	5′-AGCCGCCAGATCCCCGCA-3′
	Reverse	5′-GCCGAAGGAGTTCCAGTTGT-3′
*MiR-506*	Forward	5′-GCGGCTTTGTGCTTGATCTAA-3′
	Reverse	5′-GTGCAGGGTCCGAGGT-3′

### Cell Culture

The four human CRC cell lines HCT116, LoVo, HT29, and SW480 were purchased from the Cell Bank of the Chinese Academy of Sciences (Shanghai, China). HCT116 cells and HT29 cells were grown in McCOY’s 5A medium, LoVo cells were grown in F12K medium, and SW480 cells were grown in L-15 medium (Gibco, Carlsbad, CA, United States) containing 10% fetal bovine serum (FBS) (Gibco), and cells were incubated at 37°C with 5% CO_2_.

### Screening of Cell Lines

Cells from each cell line were plated on six-well plates (2 × 10^5^ cells per well) and incubated for 48 h to achieve a cell density of 80%. One milliliter of TRIzol reagent was added to lyse the cells. RNA was extracted with TRIzol reagent (TransGen Biotech, Beijing, China). *MiR-506* and *UHRF1* mRNA expression levels were detected by qRT-PCR. The assays were performed in triplicate.

### Vector Construction and Cell Infection

*Pre-miR-506* lentivirus, *miR-506* inhibitor lentivirus, *UHRF1*-overexpressing lentivirus, *sh-UHRF1* lentivirus, *KISS1*-overexpressing lentivirus and *PI3K*-overexpressing lentivirus were purchased from GeneChem (Shanghai, China). Infection was performed using polybrene (GeneChem). A lentivirus infection efficiency of more than 80% was considered successful. According to the manufacturer’s instructions, the multiplicity of infection for HCT116 cells was 10, and the multiplicity of infection for SW480 cells was 30. After 48 h of infection, the cells were digested for further cell culture. All experiments were performed according to the manufacturer’s instructions.

### Detection of *miR-506*, *UHRF1*, and *KISS1* mRNA Expression

Cells from each cell line were plated on six-well plates (2 × 10^5^ cells per well) and then incubated for 48 h to achieve a cell density of 80%. One milliliter of TRIzol reagent was added to lyse the cells. Then, *miR-506*, *UHRF1*, and *KISS1* mRNA expression levels were analyzed by qRT-PCR. All assays were performed in triplicate.

### Western Blot Analysis

Radioimmunoprecipitation assay buffer was added to each group of transfected cells for protein extraction, and approximately 60 μg of total protein was loaded onto an 8% SDS-PAGE gel (Beyotime) and transferred to a PVDF membrane at 300 mA for 1.5 h. The PVDF membrane was blocked with 5% skim milk for 2 h at room temperature and then incubated with anti-*UHRF1*, anti-*KISS1* or anti-*GAPDH* (1:1000; Sigma, St. Louis, MO, United States) and anti-*p*-*PI3K*, anti-*AKT*, anti-*p*-*AKT*, anti-*NF-*κ*B* (*p65*) or anti-*MMP9* (1:1000; Abcam, United States) antibodies overnight at 4°C. After washing, the membrane was incubated with HRP-conjugated goat anti-mouse IgG (1:5000; Beyotime) at room temperature for 90 min, washed three times in PBS and then visualized using ECL reagent. All assays were performed in triplicate.

### Proliferation Assay

Each group of transfected cells (1 × 10^5^ cells per well) was seeded in 24-well plates and then incubated for 24 h at 37°C and 5% CO_2_. The reagent 5-ethynyl-2′-deoxyuridine (EdU; Beyotime, Haimen, China) was added to each well, and then, the cells were incubated for another 2 h. The cells were subsequently fixed with paraformaldehyde and stained with 4,6-diamidino-2-phenylindole (DAPI) and Alexa Fluor 555 azide (Beyotime). Proliferating cells were stained red with Alexa Fluor 555 azide, and all nuclei were stained blue with DAPI. Five fields of view were randomly photographed under a microscope for statistical analysis and measurement. The statistical method used was as follows: cell proliferation rate = number of proliferating cells/total number of cells. All assays were performed in triplicate.

### Migration Assay

Transwell chambers (Corning, NY, United States) were used for the migration assay. Infected HCT116 cells (6 × 10^4^ cells per well) or SW480 cells (9 × 10^4^ cells per well) were suspended in serum-free culture medium and seeded into the upper chamber, and 800 μl of complete medium was added to the lower chamber. After incubation for 48 h at 37°C and 5% CO_2_, the cells were fixed with paraformaldehyde and stained with crystal violet. Five random fields of view were photographed under a microscope for statistical analysis and measurements. Images were obtained using an Olympus CX41 microscope (Nikon, Tokyo, Japan), and the number of cells in different treatment groups was assessed by manual counting. All assays were performed in triplicate.

### Invasion Assay

Transwell chambers (Corning, NY, United States) were used for the invasion assays. Matrigel (100 μl) (Becton Dickinson, Franklin Lake, NJ, United States) was placed into the upper chamber. Transfected HCT116 cells (9 × 10^4^ cells per well) or SW480 cells (12 × 10^4^ cells per well) were suspended in serum-free culture medium and seeded into the upper chamber, and 800 μl of complete medium was added to the lower chamber. After incubation for 48 h at 37°C and 5% CO_2_, the cells were fixed with paraformaldehyde and stained with crystal violet. Five random fields of view were photographed under a microscope for statistical analysis and measurements. Images were obtained using an Olympus CX41 microscope (Nikon, Tokyo, Japan), and the number of cells in different treatment groups was assessed by manual counting. All assays were performed in triplicate.

### Target Gene Screening Assay

TargetScan^1^, MiRanda^2^, and PicTar^3^ online tools were applied to jointly predict miRNAs that bind to *UHRF1*. Through predictive analysis, we obtained four miRNAs, of which *miR-124-3p* and *miR-9-5p* had been previously reported, and we therefore excluded them from our analysis. We found that the function of *miR-2836* had not been reported in the literature, so it was also excluded from our analysis. As *miR-506* is known to act as a tumor suppressor in other cancer tissues, we speculated that *UHRF1* may be the target of *miR-506*.

### Luciferase Assay

*MiR-506* mimics and *miR-506* negative control vectors were purchased from GenePharma (Shanghai, China). The *UHRF1* wild-type vector with a potential binding sequence and the mutant vector were purchased from GeneChem (Shanghai, China). Transfection was performed using Lipofectamine 3000 (Invitrogen). HCT116 cells transfected with *miR-506* mimics (5′-UAAGGCACCCUUCUGAGUAGA-3′, 5′-UACUCAGAAGGGUGCCUUAUU-3′) or miR-506 negative control vectors (sense 5′-UUCUCCGAACGUACGUTT-3′, antisense 5′-ACGUGACACGUUCGGAGAATT-3′) were cultured for 24 h. Firefly/Renilla luciferase activity was used as an internal control. The wild-type vector and mutant vector (1 μg) were transfected into the cells, and the cells were collected after 48 h of culture. A fluorescein assay kit (Beyotime) was used to extract fluorescein from each group, and then, the fluorescence level of each group was determined by a multi-function microplate reader. All assays were performed in triplicate.

### Tumor Formation in a Nude Mouse Model

Athymic male BALB/c nude mice (SLAC, Shanghai, China) were bred in the absence of specific pathogens. The trial protocol was approved by the Experimental Animal Ethics Committee of Fujian Medical University. MiR-506-overexpressing cells and vector control cells were trypsinized, and then, the cells were resuspended in medium at a concentration of 3 × 10^7^ cells/ml. HCT116 cells (0.2 ml) were injected subcutaneously into the left flanks of 5-week-old mice (4 mice per group). *MiR-506* inhibitor cells and the vector control cells were trypsinized, and then, the cells were resuspended in medium at a concentration of 3 × 10^7^ cells/ml. SW480 cells (0.2 ml) were injected subcutaneously into the left flank of 5-week-old mice (4 mice per group). The subcutaneously growing tumors were evaluated twice weekly after transplantation. The mice were sacrificed 4 weeks (HCT116 cells) or 6 weeks (SW480 cells) later, and the weights of the subcutaneous tumors were recorded. The tissues were embedded in paraffin, sectioned, and then stained to determine the protein expression of *UHRF1*, *KISS1*, *p-PI3K*, *NF-*κ*B*, and *MMP9* via immunohistochemistry.

### Statistical Analysis

Data were statistically analyzed using SPSS 16.0 software. Quantitative data were analyzed by Student’s *t*-test, and the results are expressed as the mean ± SD. The results of immunohistochemistry were tested by an independent sample χ^2^ test. *p* < 0.05 was considered significant.

## Results

### *UHRF1* Is Highly Expressed in CRC Tissues Compared to Adjacent Normal Tissues

**FIGURE 1:**
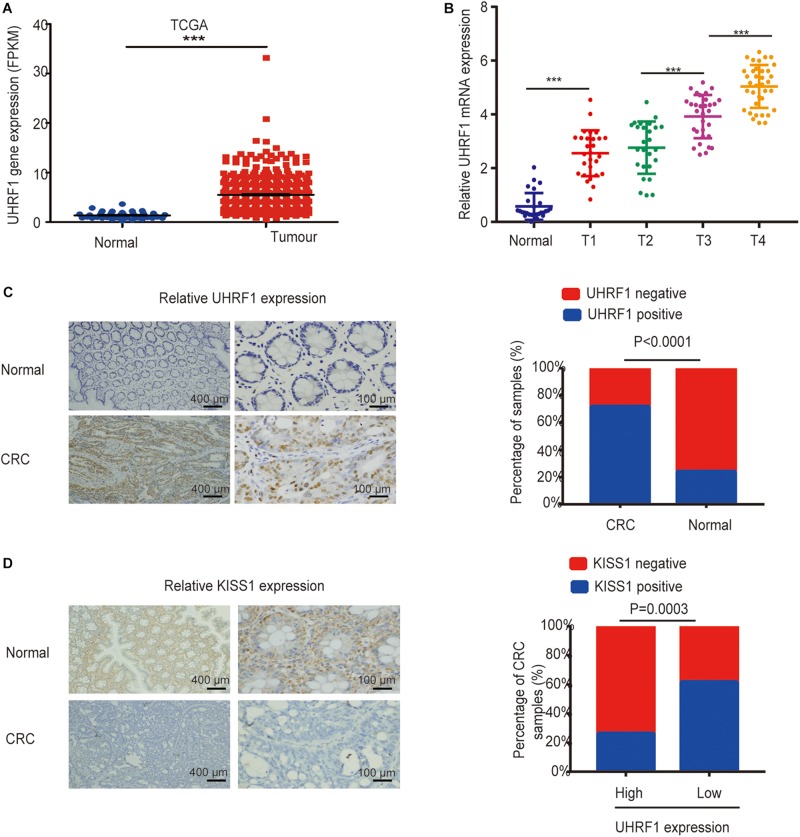
Expression of *UHRF1* and *KISS1* in CRC. **(A)** Expression of *UHRF1* mRNA in CRC and adjacent normal tissues from the TCGA database. **(B)** The expression level of *UHRF1* mRNA in tissues was detected by qRT-PCR. **(C)** Immunohistochemistry results showed that *UHRF1* protein expression was significantly different between cancer tissues and adjacent normal tissues. **(D)** Immunohistochemistry showed that *UHRF1* protein and *KISS1* protein expression were negatively correlated in colorectal cancer. ^∗∗∗^*p* < 0.001.

In our investigations into the expression of *UHRF1* in CRC, we found that *UHRF1* was highly expressed in CRC according to The Cancer Genome Atlas (TCGA) database (*p* < 0.001; Figure 1A). Next, we collected human CRC specimens (T1, *n* = 28; T2, *n* = 26; T3, *n* = 31; and T4, *n* = 36) and adjacent normal tissues (*n* = 27) and examined the expression of *UHRF1* in each sample by qRT-PCR. The results showed that the expression of *UHRF1* was significantly higher in T1 than in adjacent normal tissues (*p* < 0.001; Figure 1B). Additionally, no significant difference in *UHRF1* expression was found between T1 and T2 (*p* = 0.356); *UHRF1* expression in T4 and T3 was significantly higher than that in T2 (*p* < 0.001; Figure 1B), but *UHRF1* expression was significantly higher in T4 than in T3 (*p* < 0.001; Figure 1B). The immunohistochemistry results revealed that among CRC tissues, 87 cases were positive for *UHRF1* expression, and 34 cases were negative for *UHRF1* expression; among the adjacent normal tissues, 29 cases were positive for *UHRF1* expression, and 92 cases were negative for *UHRF1* expression. Altogether, these results demonstrate that *UHRF1* expression is significantly higher in CRC than in adjacent normal tissues (*p* < 0.001; Figure 1C and Table 2).

**TABLE 2:** Relationship between the *UHRF1* levels and clinicopathological features of 121 patients with CRC.

**Variable**		***n***	***UHRF1* level**		***p***
				
			**Low**	**High**	
Age	<60	58	13	45	0.1819
	≥60	63	21	42	
Sex	Male	69	23	46	0.1401
	Female	52	11	41	
Histological grade	Well	37	14	23	0.1137
	Moderate, poor	84	20	64	
Depth of invasion	T1 + T2	54	22	32	0.0055
	T3 + T4	67	12	55	
*KISS1* level	Low	77	13	64	0.0003
	High	44	21	23	

### *UHRF1* Inhibits *KISS1* Expression in CRC

**FIGURE 2:**
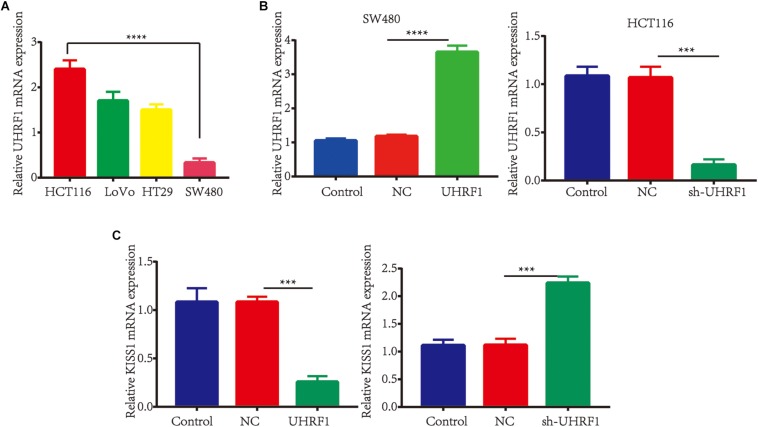
*UHRF1* inhibits *KISS1* mRNA expression. **(A)** qRT-PCR was used to detect the expression of *UHRF1* mRNA in four colorectal cancer cell lines. The expression of *UHRF1* mRNA in HCT116 cells was significantly higher than that in SW480 cells (^∗∗∗∗^*p* < 0.0001). **(B)** SW480 and HCT116 cells were infected with *UHRF1*-overexpressing and knockdown lentivirus. The expression of *UHRF1* mRNA and protein in each group was detected by qRT-PCR. **(C)** The overexpression of *UHRF1* significantly inhibited the expression of *KISS1* mRNA, and the knockdown of *UHRF1* expression significantly promoted *KISS1* mRNA expression. ^∗∗∗^*p* < 0.001, ^∗∗∗∗^*p* < 0.0001.

To investigate the association between the *UHRF1* and *KISS1* proteins in CRC, immunohistochemistry was used to detect the expression of *KISS1* in 121 CRC specimens. The results showed that in 87 CRC tissues with high UHRF1 protein expression, 23 cases were positive for *KISS1* protein expression, and 64 cases were negative. Among the 34 cases of CRC with low levels of *UHRF1* protein, 21 and 13 cases were positive and negative, respectively, for *KISS1* protein expression. Thus, *UHRF1* protein and *KISS1* protein expression were found to be negatively correlated in CRC (*p* < 0.0001; Figure 1D and Table 2). Next, we investigated whether *UHRF1* inhibits *KISS1* expression. We detected the expression of *UHRF1* mRNA in four colorectal cell lines (HCT116, LoVo, HT29, and SW480) by qRT-PCR. The results showed that the expression of *UHRF1* mRNA was highest in HCT116 cells and lowest in SW480 cells (Figure 2A). SW480 cells were infected with UHRF1-overexpressing lentivirus, and HCT116 cells were infected with *sh-UHRF1* lentivirus. The results of the qRT-PCR analyses showed that *UHRF1* expression was significantly higher in the *UHRF1*-overexpressing group than in the negative control group and that the expression of *UHRF1* was significantly lower in the *sh-UHRF1* group than in the negative control group (Figure 2B), confirming that the cells were successfully infected. *KISS1* mRNA expression was decreased in the *UHRF1*-overexpression group, while it was increased in the *sh-UHRF1* group (Figure 2C).

### *UHRF1* Promotes the Proliferation, Migration, and Invasion of CRC Cells by Inhibiting *KISS1*-Induced Activation of the *PI3K/NF-*κ*B* Signaling Pathway

**FIGURE 3:**
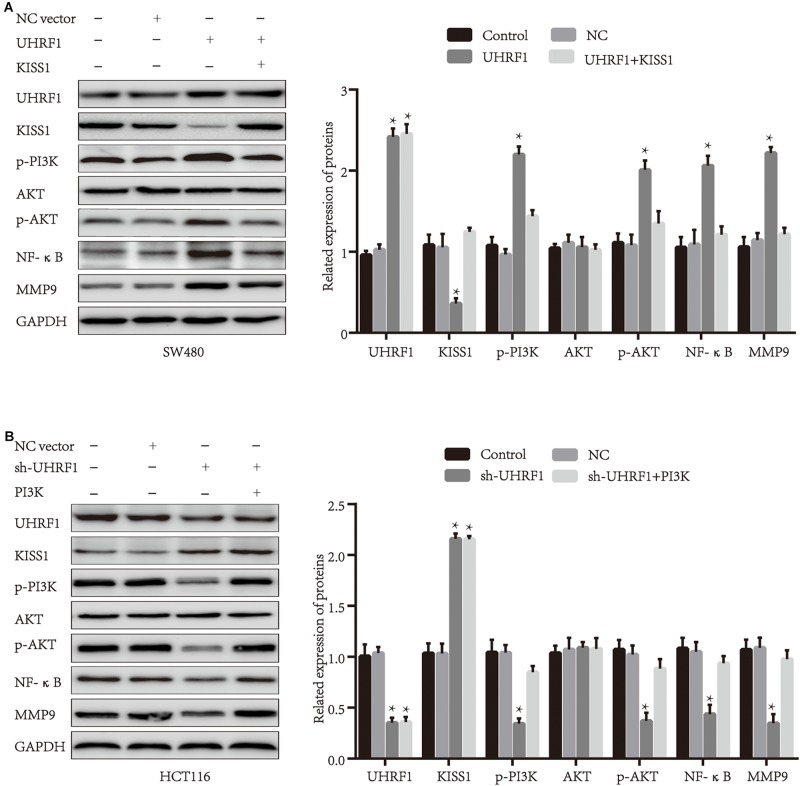
*UHRF1* activates the *PI3K/NF-*κ*B* signaling pathway by inhibiting *KISS1* protein expression. **(A)** *UHRF1* overexpression inhibits *KISS1* protein expression and activates *PI3K/NF-*κ*B* signaling pathway-related proteins. When *KISS1* protein is activated, *PI3K/NF-*κ*B* signaling axis-related protein expression levels are reversed. **(B)** Similarly, the knockdown of *UHRF1* promotes *KISS1* protein expression and inhibits *PI3K/NF-*κ*B* signaling pathway-related proteins. When *PI3K* protein is activated, *PI3K/NF-*κ*B* signaling axis-related protein expression levels are reversed. ^∗^*p* < 0.05.

**FIGURE 4:**
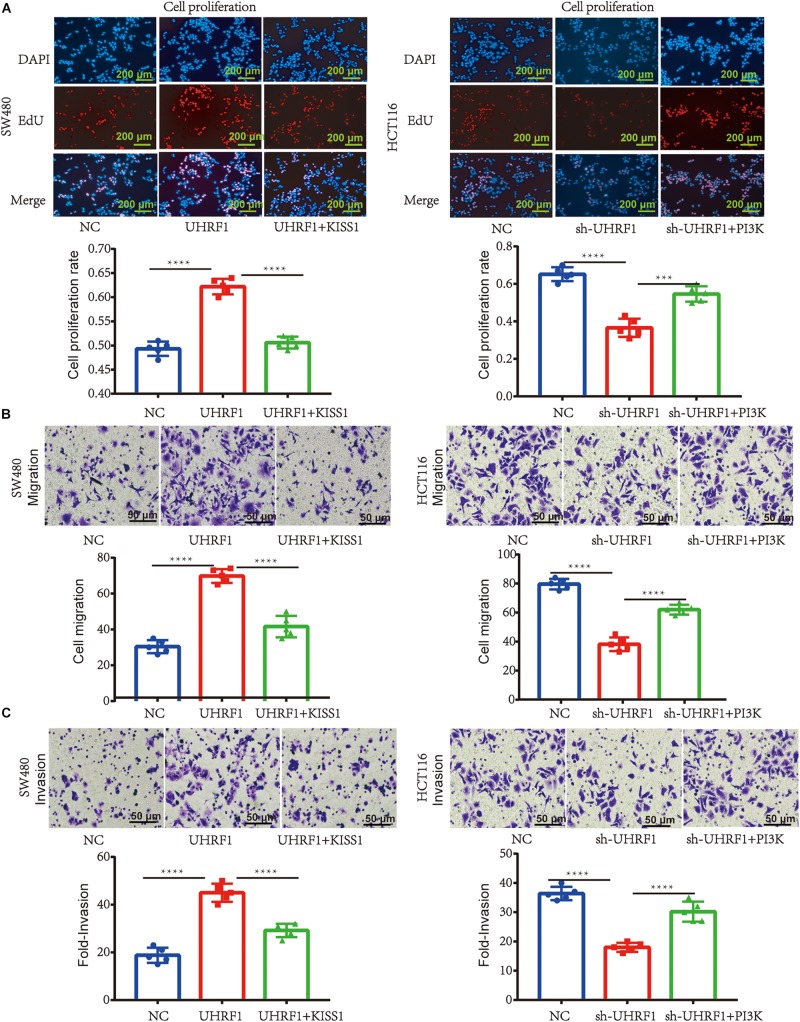
*UHRF1* promotes the proliferation, migration, and invasion of CRC through the *KISS1/PI3K/NF-*κ*B* signaling axis. **(A)** The proliferation of cells in each group is shown. **(B)** The migration of cells in each group is shown. **(C)** The invasion of each group of cells is shown. This result demonstrates that *KISS1* overexpression reverses the proliferation, migration, and invasion of CRC cells transfected with *UHRF1*-overexpressing lentivirus. *sh-UHRF1* inhibits the proliferation, migration, and invasion of colorectal cancer cells in HCT116 cells; the overexpression of *PI3K* reverses the proliferation, migration, and invasion of CRC cells transfected with *sh-UHRF1*. ^∗∗∗^*p* < 0.001, ^∗∗∗∗^*p* < 0.0001.

Next, we explored the mechanism of *UHRF1* in CRC metastasis. Western blotting results revealed that the overexpression of *UHRF1* inhibits *KISS1* protein expression and promotes the expression of *PI3K/NF-*κ*B* signaling pathway-related proteins; when we activated KISS1 protein expression in cells infected with the *UHRF1* overexpression vector, the expression of PI3K/NF-κB signaling pathway-associated proteins was subsequently inhibited (Figure 3A). Infection of CRC cells with *sh-UHRF1* promoted the expression of the *KISS1* protein and inhibited the expression of *PI3K/NF-*κ*B* signaling pathway-related proteins. When we activated the expression of *PI3K* protein in cells infected with the *sh-UHRF1* vector, the expression of the *PI3K/NF-*κ*B* signaling pathway-associated proteins was then activated (Figure 3B). This result revealed that *UHRF1* activates the *PI3K/NF-*κ*B* signaling pathway by inhibiting *KISS1* in CRC. To further explore whether this mechanism is involved in the malignant behavior of CRC, we performed proliferation, migration, and invasion assays and further verified that *UHRF1* promotes the proliferation, migration, and invasion of CRC; however, the activation of *KISS1* reversed this trend. Additionally, *sh-UHRF1* inhibited the proliferation, migration and invasion of CRC, but the activation of *PI3K* reversed this trend (Figures 4A–C). Therefore, the results of this study revealed that *UHRF1* inhibits the proliferation, migration, and invasion of CRC by inhibiting *KISS1*-induced activation of the *PI3K/NF-*κ*B* signaling pathway.

### *MiR-506* Is Expressed at Low Levels in CRC and Targets *UHRF1*

**FIGURE 5:**
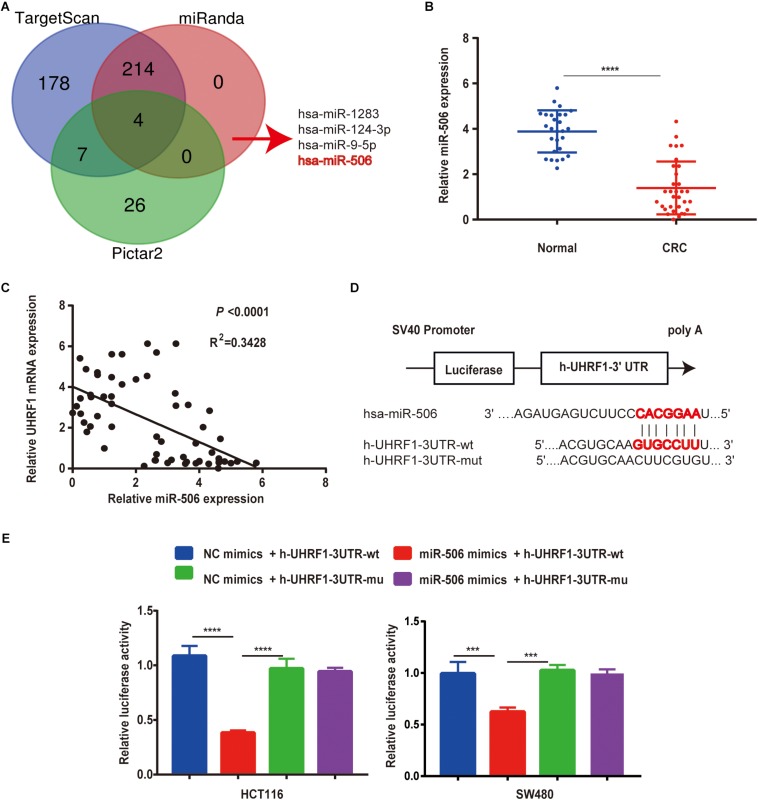
*MiR-506* targets *UHRF1* and is negatively correlated with *UHRF1* expression in CRC. **(A)** Three databases were used to jointly predict miRNAs that may bind to *UHRF1*. **(B)** The expression level of *miR-506* in adjacent normal tissues (*n* = 27) was significantly higher than that in CRC tissues (*n* = 32). **(C)** A negative correlation was found between *miR-506* and *UHRF1* expression in CRC. **(D)** The prediction of potential binding sites for *miR-506* and *UHRF1* by bioinformatics and the design of mutant vector sequences. **(E)** WT or MU *UHRF1* luciferase constructs were transfected into HCT116 cells and SW480 cells with the *miR-506* mimics or *miR-NC*, and luciferase activity was detected 48 h later. *MiR-506* reduced the intensity of the luciferase-*UHRF1* reporter vector in HCT116 cells (^∗∗∗∗^*p* < 0.0001) and SW480 cells (^∗∗∗^*p* < 0.001).

To further explore the mechanism of *UHRF1* expression in CRC, this study used data from three databases to predict miRNAs that might bind to *UHRF1*. The results revealed that four miRNAs (*miR-1283*, *miR-506*, *miR-9-5p*, and *miR-124-3p*) may bind to *UHRF1* (Figure 5A). A review of the literature revealed that low *miR-506* expression has been reported in a variety of tumors. Moreover, by qRT-PCR, we found that *miR-506* was downregulated in CRC and negatively correlated with *UHRF1* mRNA expression (Figures 5B,C). Potential binding sites were predicted by the TargetScan database (Figure 5D). Finally, the results of a luciferase reporter assay revealed that h-*UHRF1*-WT significantly inhibited luciferase expression in HCT116 cells and SW480 cells, whereas h-*UHRF1*-MU failed to inhibit luciferase expression (Figure 5E).

### *MiR-506* Inhibits CRC Proliferation, Migration, and Invasion via the *UHRF1/KISS1* Signaling Axis

**FIGURE 6:**
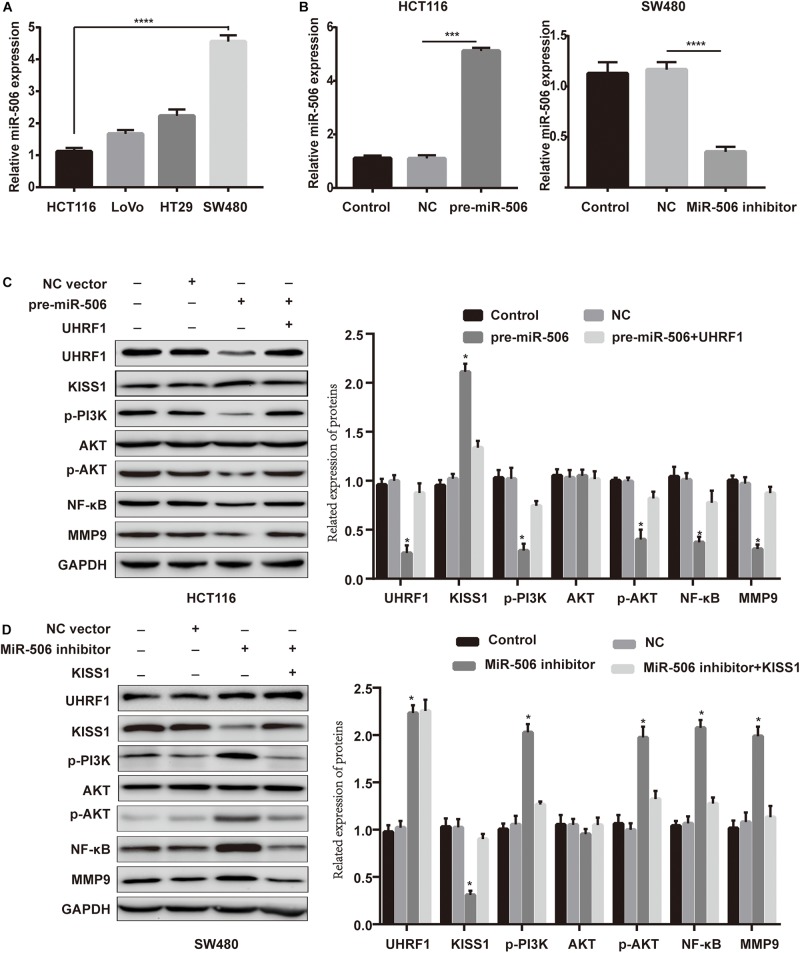
*MiR-506* activates *KISS1* expression and inhibits the *PI3K/NF-*κ*B* signaling axis by targeting *UHRF1*. **(A)** qRT-PCR was used to detect the expression level of *miR-506* in four CRC cells. **(B,C)** HCT116 cells and SW480 cells were infected with *pre-miR-506* lentivirus and *miR-506* knockdown lentivirus. The expression level of *miR-506* in each group was examined by qRT-PCR. **(C)** *Pre-miR-506* activated KISS1 to repress *PI3K/NF-*κ*B* signaling pathway-associated proteins by inhibiting *UHRF1* protein; when *UHRF1* protein was activated, *KISS1/PI3K/NF-*κ*B* signaling axis-related protein expression levels were reversed. **(D)** The *miR-506* inhibitor inhibited *KISS1* activation of *PI3K/NF-*κ*B* signaling pathway-related proteins by promoting *UHRF1* protein expression; when *KISS1* protein was activated, *PI3K/NF-*κ*B* signal transduction axis-related protein expression levels were reversed. ^∗^*p* < 0.05, ^∗∗∗^*p* < 0.001, ^∗∗∗∗^*p* < 0.0001.

**FIGURE 7:**
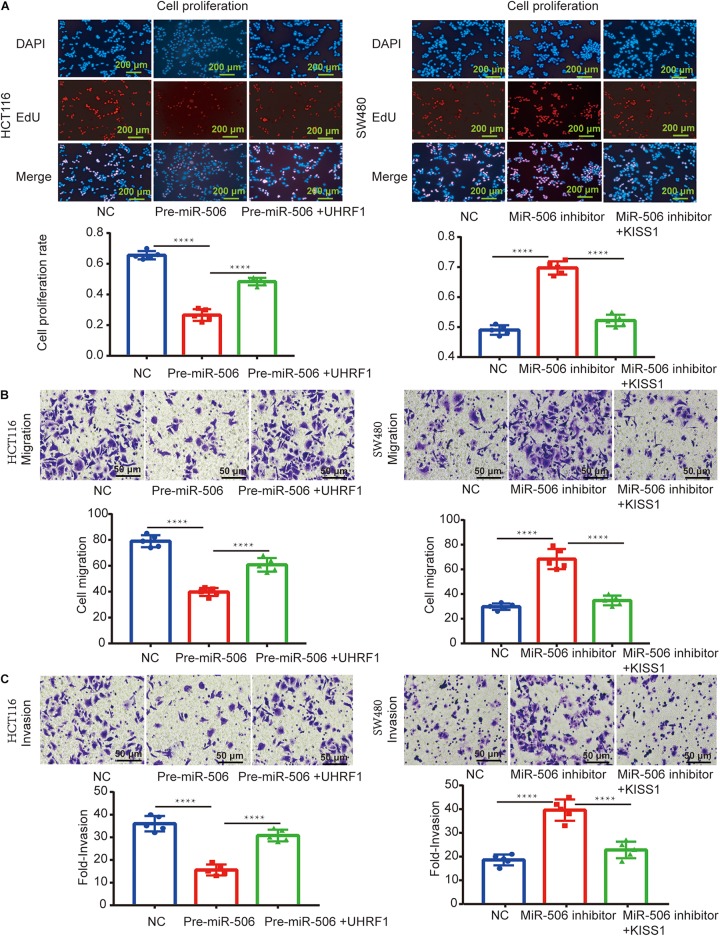
*MiR-506* inhibits the proliferation, migration, and invasion of CRC through the *UHRF1/KISS* signaling axis. **(A–C)** *Pre-miR-506* inhibits the proliferation, migration, and invasion of HCT116 cells. *UHRF1* overexpression reversed the proliferation, migration, and invasion of CRC cells infected with the *pre-miR-506* lentivirus. The *miR-506* inhibitor promoted the proliferation of SW480 cells. Regarding migration and invasion, the overexpression of *KISS1* reversed the proliferation, migration, and invasion of CRC cells transfected with the *miR-506* inhibitor. ^∗∗∗∗^*p* < 0.0001.

Next, we investigated whether *miR-506* targets *UHRF1* in CRC and found that the proliferation, migration, and invasion of CRC are affected by the *KISS1/PI3K/NF-*κ*B* signaling axis. We detected the expression of *miR-506* in four colorectal cell lines (HCT116, LoVo, HT29, and SW480) by qRT-PCR. The results showed that the expression of *miR-506* was highest in SW480 cells and lowest in HCT116 cells (Figure 6A). Therefore, this study used *miR-506* knockdown lentivirus to infect SW480 cells and infected HCT116 cells with *pre-miR-506* lentivirus. The qRT-PCR results showed that *miR-506* expression was significantly higher in the *pre-miR-506* group than in the negative control group and significantly lower in the *miR-506* inhibitor group than in the negative control group (Figure 6B). These results confirmed that the cells were successfully infected with lentivirus. The Western blotting results further showed that *pre-miR-506* inhibited the expression of the *UHRF1* protein and activated *KISS1* expression to suppress the expression of *PI3K/NF-*κ*B* signaling pathway-related proteins. When the expression of *UHRF1* was activated, the expression of the *KISS1/PI3K/NF-*κ*B* signaling axis was reversed. The *miR-506* inhibitor led to the opposite effect (Figures 6C,D). Finally, the results of the proliferation, migration, and invasion experiments further confirmed that *miR-506* inhibited the proliferation, migration, and invasion of CRC and that the activation of *UHRF1* reversed this trend (Figures 7A–C).

### *MiR-506* Inhibits Cell Proliferation and Invades CRC Cells in Xenograft Nude Mice

**FIGURE 8:**
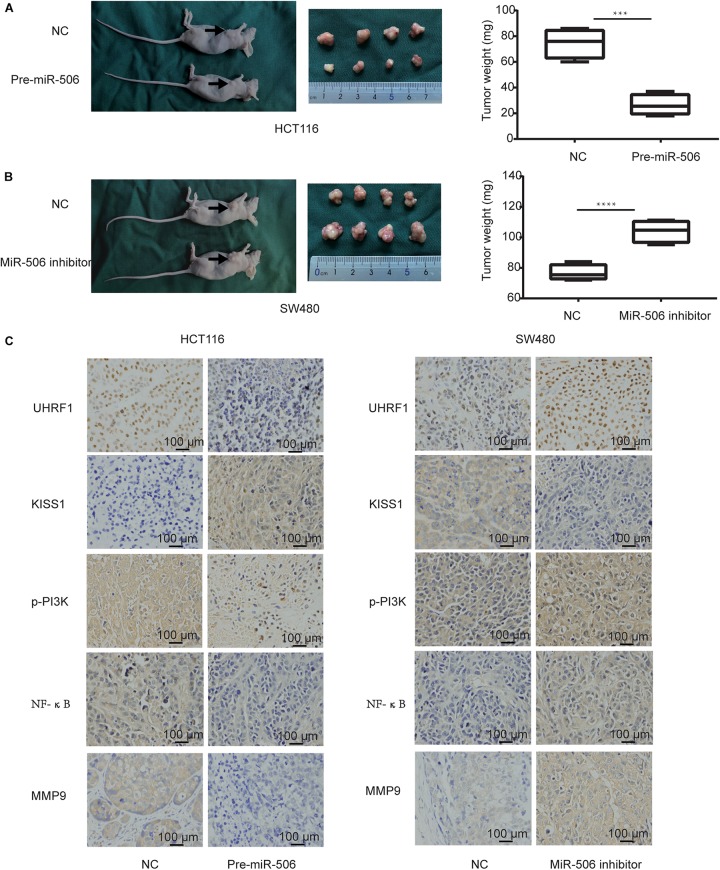
*MiR-506* inhibits the proliferation and invasion of CRC *in vivo*. **(A)** *Pre-miR-506* significantly inhibited CRC proliferation compared with the negative control group. **(B)** The *miR-506* inhibitor significantly promoted CRC proliferation compared with the negative control group. **(C)** Immunohistochemistry was used to detect the expression of *UHRF1*, *KISS1*, *p-PI3K*, *NF-*κ*B*, and *MMP9* proteins in each group of tumors. Compared with the negative control group, *pre-miR-506* significantly promoted *KISS1* protein expression and inhibited *UHRF1*, *p-PI3K*, *NF-*κ*B*, and *MMP9* protein expression. Compared with the negative control group, the *miR-506* inhibitor significantly promoted *UHRF1*, *p-PI3K*, *NF-*κ*B*, and *MMP9* protein expression and inhibited *KISS1* protein expression. ^∗∗∗^*p* < 0.001, ^∗∗∗∗^*p* <0.0001.

Finally, we further verified the previous results through *in vivo* experiments. Compared with the negative control group, CRC proliferation was significantly inhibited in the *miR-506* group (Figure 8A), and CRC proliferation was significantly enhanced in the *miR-506* inhibitor group (Figure 8B). Immunohistochemistry revealed that *miR-506* inhibits *UHRF1*, *p-PI3K*, *NF-*κ*B*, and *MMP9* protein expression and promotes *KISS1* protein expression in xenograft nude mice. In contrast, the *miR-506* inhibitor promotes *UHRF1*, *p-PI3K*, *NF-*κ*B*, and *MMP9* protein expression and inhibits *KISS1* protein expression (Figure 8C).

## Discussion

Colorectal cancer is a type of malignant tumor with high invasive and metastatic abilities. CRC is currently ranked third among all cancer deaths, and CRC metastasis is an important factor in increasing mortality (Bray et al., 2018). Although multimodal treatments improve the prognosis of CRC (Ganesh et al., 2019; Li S. et al., 2019; Siravegna et al., 2019), the distant metastasis of cancer cells remains the chief culprit of treatment failure. Therefore, a better understanding of the mechanisms of CRC metastasis is essential for the development of new therapeutic strategies.

The occurrence and development of CRC is accompanied by the activation of proto-oncogenes and the loss of tumor suppressor genes (Sanz-Garcia et al., 2017; Zhang and Shay, 2017). DNA methylation has been found to play an important role in the inactivation of tumor suppressor genes (Schubeler, 2015; Li Z. et al., 2019), and studies have shown that DNA methylation requires the involvement of *UHRF1* (Nishiyama et al., 2013; Ferry et al., 2017; Li et al., 2018b). High expression of *UHRF1* in tumor tissues has been reported to promote tumor metastasis (Oh et al., 2018; Hu et al., 2019). Related studies have also found that *UHRF1* is highly expressed in breast cancer, bladder cancer, prostate cancer, and CRC (Jenkins et al., 2005; Zhu et al., 2015; Wan et al., 2016; Saidi et al., 2017). In this study, *UHRF1* was found to be highly expressed in CRC tissues through the TCGA database. The expression of *UHRF1* in T1- and T2-stage tumors was significantly higher than that in adjacent normal tissues but significantly lower than that in T3- and T4-stage tumors. In addition, *UHRF1* overexpression was found to promote the proliferation, migration and invasion of CRC, which suggests that the high expression of *UHRF1* in CRC is closely related to CRC initial and development. However, the specific mechanism by which *UHRF1* regulates CRC metastasis remains unclear.

The *KISS1* gene is an important tumor suppressor. The loss of *KISS1* expression in CRC has been reported (Chen et al., 2014); *KISS1* expression is low in various tumor tissues, which can enhance the growth, invasion, and migration of tumor cells (Chen et al., 2016). In this study, immunohistochemical analysis revealed that *KISS1* protein expression in CRC was negatively correlated with *UHRF1* expression. Moreover, *UHRF1* overexpression promotes CRC proliferation, migration, and invasion by inhibiting *KISS1* gene expression and activating the *PI3K/NF-*κ*B* signaling pathway. Conversely, knockdown of *UHRF1* expression can promote *KISS1* gene expression to block the *PI3K/NF-*κ*B* signaling pathway, inhibiting the proliferation, migration, and invasion of CRC.

The aim of gene-targeted therapy is the design of a therapeutic drug that is appropriate at the cellular and molecular level and that exhibits specificity to a well-defined carcinogenic site. After entering the body, the drug should target a carcinogenic site and cause tumor cell-specific death. Previous studies have found that the drug targeting- and drug encapsulation-related challenges in designing targeted drugs are the high molecular weight and the difficulty in encapsulating these drugs to keep them stable in body fluids prior to reaching the target cancer cells.

MiRNAs are a class of non-coding RNAs that are approximately 22 nucleotides in length. These molecules play an important role in tumor metastasis because they are involved in tumor cell proliferation, apoptosis, invasion, and autophagy (Chipman and Pasquinelli, 2019). Previous studies have shown that in a variety of tumor tissues, targeted binding of miRNAs to mRNAs leads to gene silencing and can thus affect the biological behavior of tumor cells (Farazi et al., 2013). Related studies have found that miRNAs can be encapsulated into nanoparticles by exosomes, which can remain stable in the blood and that miRNAs encapsulated by exosomes can affect the metastatic ability of cancer cells in nude mice. MiRNAs have the advantages of their small molecular weights and ease of packaging and are currently being researched for targeted therapy. Therefore, miRNAs are highly promising for targeted therapy. Studies have shown that *miR-92a-3p* is upregulated in CRC and promotes the migration of CRC by targeting *NF2* (Alcantara and Garcia, 2019). *MiR-452* activates *Wnt/*β-catenin to promote CRC metastasis (Li et al., 2018a). In addition to upregulated miRNAs, downregulated miRNAs such as *miR-144*, *miR-548c-5p*, and *miR-198* also play important roles in tumorigenesis. *MiR-144* has been reported to target *GSPT1* to inhibit the metastasis of CRC (Xiao et al., 2015), and *miR-548c-5p* has been found to act as a tumor suppressor in CRC by targeting *PGK1* (Ge et al., 2019). The above results indicate that dysregulated miRNA expression has been observed in CRC and that the abnormal expression of specific miRNAs is associated with metastasis and prognosis in CRC.

The results of our study revealed that the expression of *UHRF1* in CRC is closely related to pathological stage (Figure 1B) and that *UHRF1* can promote the metastasis of CRC (Figure 4), but a miRNA targeting *UHRF1* expression in CRC has not yet been reported. Through bioinformatics analysis, we found that *miR-506* has a potential binding site on *UHRF1*. Related studies have reported that *miR-506* targets *ZEB2* to inhibit gastric cancer invasion and is associated with the poor prognosis of gastric cancer (Wang et al., 2019). In pancreatic cancer, the overexpression of *miR-506* blocks the *SPHK1/AKT/NF-*κ*B* signaling pathway and inhibits pancreatic cancer metastasis (Li et al., 2016). However, the expression of *miR-506* in CRC and its mechanism of action are still unclear. We detected *miR-506* in CRC and adjacent normal tissues by PCR and found that *miR-506* was downregulated in normal tissues adjacent to cancer tissues. In addition, the expression of *miR-506* was negatively correlated with the expression of *UHRF1*, suggesting that *UHRF1* is the target molecule of *miR-506*. Luciferase reporter assays confirmed that *miR-506* targets *UHRF1*. Then, we performed Western blotting and cell proliferation assays. Migration and invasion assays further confirmed that *miR-506* targets *UHRF1* to inhibit the proliferation, migration, and invasion of CRC cells via the *KISS1/PI3K/NF-*κ*B* signaling axis. The opposite results were found with the knockdown of *miR-506*. Finally, through *in vivo* ectopic tumor formation experiments, we further confirmed that *miR-506* targets *UHRF*1 to inhibit the proliferation and invasion of CRC by the *KISS1/PI3K/NF-*κ*B* signaling pathway.

Current studies in the literature have indicated that *UHRF1*, as an oncogene in CRC, activates the *PI3K/NF-*κ*B* signaling pathway by inhibiting the expression of *KISS1* to promote tumorigenesis and progression. In this work, the expression of *UHRF1* was found to be regulated by *miR-506*, which affects the biological behavior of CRC. As precision medicine continues to advance, gene-targeted therapy is an important next step. MiRNA-based targets are promising and may be important potential molecules in future targeted therapies for CRC.

## Data Availability Statement

We declare that the materials described in the manuscript, including all relevant raw data, will be freely available to any scientist wishing to use them for non-commercial purposes, without breaching participant confidentiality.

## Ethics Statement

The studies involving human participants were reviewed and approved by the Ethics Committee of the First Affiliated Hospital of Fujian Medical University. The patients/participants provided their written informed consent to participate in this study. The animal study was reviewed and approved by the Ethics Committee of the First Affiliated Hospital of Fujian Medical University.

## Author Contributions

SC designed the study and provided funding for the study. YLn participated in the entire experiment, and writing and modifying the manuscript. ZC helped to solve problems throughout the experiment, and participated in the editing and revision of the manuscript. YZ, YLu, JG, and SL participated in the Western blotting experiments and statistical analysis of the data.

## Conflict of Interest

The authors declare that the research was conducted in the absence of any commercial or financial relationships that could be construed as a potential conflict of interest.

## Footnotes

1 http://www.targetscan.org/

2 http://www.microrna.org/microrna/home.do

3 http://www.pictar.org/

## Abbreviations

BSP: bisulfite sequencing PCR
CRC: colorectal cancer
DAPI: 4,6-diamidino-2-phenylindole
ECL: electrochemiluminescence
EdU: 5-ethynyl-2 ′ -deoxyuridine
PVDF: polyvinylidene fluoride
qRT-PCR: quantitative real-time PCR
RIPA: radioimmunoprecipitation assay
ROS: reactive oxygen species
UHRF1: ubiquitin-like with plant homeodomain and RING finger domains 1
3 ′ UTR: 3 ′ untranslated region.
